# RFAG-YOLO: A Receptive Field Attention-Guided YOLO Network for Small-Object Detection in UAV Images

**DOI:** 10.3390/s25072193

**Published:** 2025-03-30

**Authors:** Chengmeng Wei, Wenhong Wang

**Affiliations:** College of Computer Science, Liaocheng University, Liaocheng 252059, China; 2220170207@stu.lcu.edu.cn

**Keywords:** UAV images, YOLOv8, feature extraction, small-object detection, attention mechanism

## Abstract

The YOLO series of object detection methods have achieved significant success in a wide range of computer vision tasks due to their efficiency and accuracy. However, detecting small objects in UAV images remains a formidable challenge due to factors such as a low resolution, complex background interference, and significant scale variations, which collectively degrade the quality of feature extraction and limit detection performance. To address these challenges, we propose the receptive field attention-guided YOLO (RFAG-YOLO) method, an advanced adaptation of YOLOv8 tailored for small-object detection in UAV imagery, with a focus on improving feature representation and detection robustness. To this end, we introduce a novel network building block, termed the receptive field network block (RFN block), which leverages dynamic kernel parameter adjustments to enhance the model’s ability to capture fine-grained local details. To effectively harness multi-scale features, we designed an enhanced FasterNet module based on RFN blocks as the core component of the backbone network in RFAG-YOLO, enabling robust feature extraction across varying resolutions. This approach achieves a balance of semantic information by employing staged downsampling and a hierarchical arrangement of RFN blocks, ensuring optimal feature representation across different resolutions. Additionally, we introduced a Scale-Aware Feature Amalgamation (SAF) component prior to the detection head of RFAG-YOLO. This component employs a scale attention mechanism to dynamically weight features from both higher and lower layers, facilitating richer information flow and significantly improving the model’s robustness to complex backgrounds and scale variations. Experimental results on the VisDrone2019 dataset demonstrated that RFAG-YOLO outperformed state-of-the-art models, including YOLOv7, YOLOv8, YOLOv10, and YOLOv11, in terms of detection accuracy and efficiency. In particular, RFAG-YOLO achieved an mAP50 of 38.9%, representing substantial improvements over multiple baseline models: a 12.43% increase over YOLOv7, a 5.99% improvement over YOLOv10, and significant gains of 16.12% compared to YOLOv8n and YOLOv11. Moreover, compared to the larger YOLOv8s model, RFAG-YOLO achieved 97.98% of its mAP50 performance while utilizing only 53.51% of the parameters, highlighting its exceptional efficiency in terms of the performance-to-parameter ratio and making it highly suitable for resource-constrained UAV applications. These results underscore the substantial potential of RFAG-YOLO for real-world UAV applications, particularly in scenarios demanding accurate detection of small objects under challenging conditions such as varying lighting, complex backgrounds, and diverse scales.

## 1. Introduction

Object detection is a fundamental task in computer vision, with critical applications including military operations [1], self-driving vehicles [2], security monitoring [3], and remote sensing [4]. Recently, the analysis of UAV imagery for object detection has emerged as a vital tool across various domains, driven by the extensive coverage and high resolution of UAV data [5]. In these applications, the detection of small targets, such as vehicles, pedestrians, and critical infrastructure, plays a pivotal role in environmental monitoring, human activity analysis, and the security of essential facilities. However, accurately detecting small objects in UAV aerial imagery remains a significant challenge due to factors such as a low resolution, complex backgrounds, and scale variations.

Driven by rapid advancements in neural networks and continuous innovations by researchers worldwide, significant progress has been made in the field of object detection. These advancements have significantly enhanced the accuracy and efficiency of detection systems, while also broadening their applicability to a wide range of real-world scenarios. The deep learning-based object detection methods are broadly categorized into two main paradigms: two-stage [6] and one-stage [7] approaches. Two-stage methods generally consist of two sequential steps: (1) generating candidate regions using a selective search algorithm [8] or a Region Proposal Network (RPN) [9] and (2) extracting features from these regions and classifying them via a convolutional neural network. Prominent examples of two-stage methods include Region-Based CNN (R-CNN) [10], Fast R-CNN [11], Faster R-CNN [9], and Mask R-CNN [12]. These methods achieve a high detection accuracy by initially eliminating background regions and subsequently performing detailed analysis on the remaining candidate areas. However, their reliance on extensive computational resources often leads to slower inference speeds. In contrast, one-stage object detectors formulate the task as a regression problem, simultaneously predicting bounding box coordinates and class labels in a single forward pass through the network, offering a better balance between accuracy and efficiency. Notable examples of one-stage detectors include You Only Look Once (YOLO) [13] and the Single Shot MultiBox Detector (SSD) [14]. The SSD relies on manually defined prior boxes whose size and shape are heavily influenced by empirical settings, limiting its adaptability to diverse scenarios. Furthermore, the SSD’s reliance on low-level feature layers for prediction compromises its ability to detect small targets, resulting in lower recall rates.

Despite these advances, several critical challenges persist in UAV-based object detection. First, the limited receptive field of traditional convolutional operations struggles to capture the complex spatial relationships and fine-grained details of objects in aerial imagery, particularly for targets with irregular shapes or varying orientations. Second, the significant scale variations in UAV imagery, coupled with the presence of small objects, poses substantial difficulties for feature extraction and representation learning. Traditional multi-scale feature fusion approaches often fail to effectively balance the contribution of features from different scales, leading to suboptimal detection performance. Third, the complex and diverse backgrounds in aerial imagery, combined with varying lighting conditions and weather effects, create substantial interference that can significantly impact detection accuracy.

To address the aforementioned challenges in object detection, we propose the receptive field attention-guided YOLO (RFAG-YOLO) model, an enhanced framework based on YOLOv8. First, we introduce a novel network component, termed the receptive field network block (RFN block), which improves the model’s ability to capture fine-grained details of complex-shaped objects through dynamic adjustments of convolutional kernel weights. Second, to efficiently utilize multi-scale feature maps and optimize the trade-off between detection performance and computational efficiency, we integrate FasterNet [15] with the RFN block to construct the backbone network of RFAG-YOLO. Finally, we introduce a Scale-Aware Feature Amalgamation (SAF) module prior to the detection head. This module employs a scale attention mechanism to dynamically fuse multi-scale feature maps, thereby improving the model’s adaptability to varying resolutions and enhancing the representation of small objects. The key contributions of this study are summarized as follows:

1. The RFN block is proposed, inspired by the concept of receptive field attention (RFA) [16]. By incorporating a dynamic kernel parameter adjustment, the RFN block addresses the limitations of parameter sharing in traditional convolutions, significantly improving the ability of the RFAG-YOLO model to capture and emphasize discriminative features in local regions.
2. The RFN block is integrated with FasterNet to construct a robust backbone network. This network effectively balances semantic information across multi-resolution feature maps through staged downsampling and a hierarchical arrangement of RFN blocks, ensuring comprehensive feature representation at different scales.
3. A SAF module is introduced prior to the detection head of RFAG-YOLO. Leveraging a scale attention mechanism, the SAF module dynamically refines feature representations and significantly improves the spatial localization accuracy by adaptively weighting multi-scale features.
4. Comprehensive experiments on the VisDrone2019 dataset demonstrate that our proposed RFAG-YOLO achieves superior detection performance compared to state-of-the-art methods while maintaining a balanced trade-off between model complexity and detection accuracy. The proposed method effectively addresses the inherent challenges of UAV-based object detection, such as small object sizes, varying lighting, complex backgrounds, and diverse scales.

The remainder of this paper is organized as follows. Section 2 reviews related work in object detection, including the development of the YOLO series, YOLO-based improvements for UAV detection, and transformer-based approaches. Section 3 presents the detailed architecture of RFAG-YOLO, including the design principles and implementation details of the RFN block, the integration with FasterNet backbone, and the proposed SAF module. Section 4 describes our experimental methodology, including dataset preparation, training procedures, and results of a comprehensive evaluation against state-of-the-art object detection models on the VisDrone2019 dataset, along with ablation studies to validate the effectiveness of each proposed component. Section 5 provides a theoretical analysis of our proposed method, examines the model’s limitations, and discusses potential directions for future research. Finally, Section 6 concludes the paper.

## 2. Related Work

This section provides a comprehensive review of related work in three key areas. We begin by tracing the evolution of the YOLO series, which has revolutionized real-time object detection through continuous architectural innovations and performance improvements. We then examine recent advances in small-object detection specifically tailored for UAV imagery, focusing on various enhancement strategies and architectural modifications. The review concludes with an analysis of transformer-based object detection approaches, which represent a paradigm shift in the field but face challenges in resource-constrained UAV applications.

### 2.1. Development of YOLO Series

Due to its exceptional precision, efficiency, and practicality, the YOLO family of object detection models has established itself as a benchmark in the field since the introduction of YOLOv1 [17] in 2015. YOLOv1 revolutionized object detection by treating it as a regression problem, laying the groundwork for modern one-stage detectors. YOLOv2 [18] improved performance and stability through innovations such as Darknet-19, anchor boxes, and batch normalization, especially for small-object detection. YOLOv3 [19] enhanced the speed–precision trade-off by incorporating a feature pyramid network (FPN) [20] and multi-scale predictions, enabling detection across a wider range of object sizes.

YOLOv4 [21] achieved significant progress by integrating CSPNet [22], SPP-Block [23], the Mish activation function [24], and advanced data augmentation techniques, significantly boosting performance. YOLOv5 [25] gained widespread adoption due to its modular design, incorporating GhostNet [26] and path aggregation network (PANet) [27], as well as its user-friendly implementation. Subsequent versions, from YOLOv6 [28] to YOLOv7 [29], focused on lightweight architectures, performance optimization, and streamlined deployment.

In 2024, YOLOv9 [30] and YOLOv10 [31] were introduced. YOLOv9 introduced Programmable Gradient Information (PGI) and an auxiliary reversible branch, significantly enhancing model expressiveness and training efficiency. YOLOv10 pioneered a uniform dual-assignment strategy, eliminating the need for Non-Maximum Suppression (NMS) during training, thereby improving performance and reducing inference latency.

### 2.2. YOLO-Based Improvements for UAV Detection

Motivated by advancements in the YOLO family of object detection models, researchers have proposed numerous enhancements specifically tailored for small-target detection in UAV imagery, leading to the development of diverse drone image detection methods. Building on YOLOv5, Zeng et al. [32] introduced a hybrid coordinate attention mechanism to enhance feature extraction for small objects in UAV imagery. They further proposed an optimized bottleneck architecture to improve the discriminability between object and background features during small-target detection. Shin et al. [33] incorporated deformable convolution [34] into the YOLOv5 backbone, leveraging three kernels to simultaneously learn offsets, masks, and feature representations, thereby enhancing the extraction of fine-grained characteristics of small targets. Chen et al. [35] proposed a multi-scale feature pyramid network (SAS-FPN) to effectively integrate shallow and deep feature maps, significantly improving the model’s feature extraction capabilities. Additionally, they integrated a shuffle attention mechanism [36] into the backbone network to mitigate the impact of complex background interference. Zhu et al. [37] developed TPH-YOLOv5, a model specifically optimized for target detection in UAV-captured imagery. TPH-YOLOv5 replaces conventional prediction heads with transformer-based heads, leveraging the self-attention mechanism to enhance prediction accuracy. Furthermore, TPH-YOLOv5 incorporates the Convolutional Block Attention Module (CBAM) [38] to improve target localization in densely cluttered scenes.

### 2.3. Transformer-Based Object Detection

The emergence of transformer architectures has sparked a new wave of innovation in object detection, offering alternative approaches to traditional CNN-based methods. These transformer-based detectors have demonstrated remarkable potential in addressing long-standing challenges in object detection, particularly in handling complex spatial relationships and global context modeling. DETR [39] pioneered a paradigm shift by reformulating object detection as a direct set prediction problem, eliminating traditional hand-crafted components such as NMS and anchor generation. By leveraging a transformer encoder–decoder architecture and a set-based global loss with bipartite matching, DETR achieves competitive performance while maintaining conceptual simplicity. Building upon DETR’s success, RT-DETR [40] addresses the computational efficiency challenges by introducing a hybrid architecture that combines CNNs with transformers. Through an efficient hybrid encoder for multi-scale feature processing and uncertainty-minimal query selection, RT-DETR achieves superior performance compared to traditional YOLO models while maintaining real-time inference speeds. Specifically targeting UAV applications, Drone-DETR [41] builds upon RT-DETR by introducing an Effective Small Object Detection Network (ESDNet) and Enhanced Dual-Path Feature Fusion Attention Module (EDF-FAM), achieving significant improvements in small-object detection while maintaining a lightweight architecture.

More recently, D-FINE [42] pushed the boundaries of real-time object detection by redefining the bounding box regression task in DETR models. Through its innovative Fine-grained Distribution Refinement (FDR) and Global Optimal Localization Self-Distillation (GO-LSD) components, D-FINE achieves remarkable localization precision while maintaining real-time performance. The DEIM [43] framework further enhances transformer-based detectors by introducing Dense One-to-One matching and Matchability-Aware Loss, significantly accelerating training convergence while maintaining high accuracy. While these transformer-based approaches demonstrate impressive capabilities, their computational demands and complex architectures make them less suitable for resource-constrained UAV applications. Therefore, improving the YOLO framework remains a more practical approach for real-world drone detection scenarios.

## 3. Materials and Methods

This section delineates the architectural design and technical innovations of the proposed RFAG-YOLO model, aimed at addressing the challenges of object detection in UAV imagery, such as small object sizes and complex backgrounds. After presenting the network structure of YOLOv8, we provide the general architecture of the proposed RFAG-YOLO. Then, based on YOLOv8 as the baseline framework, three key innovations are proposed: (1) the RFN block incorporating receptive field attention, (2) an enhanced FasterNet backbone network with a hierarchical RFN block arrangement, and (3) a SAF module designed for adaptive multi-scale feature fusion.

### 3.1. Introduction to the YOLOv8 Network

**Figure 1:**
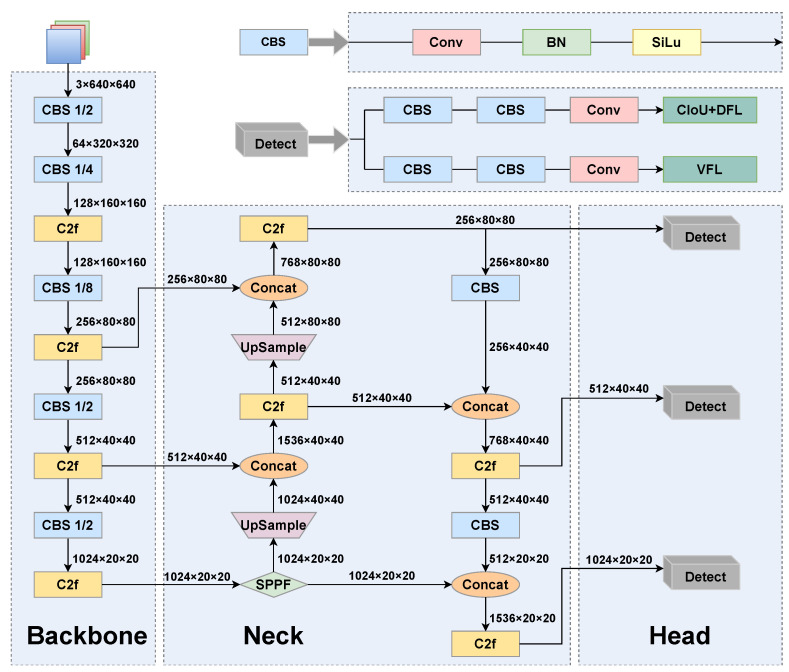
YOLOv8 network structure diagram.

In this study, we selected YOLOv8 as the baseline network for improvement due to its simple structure and stable detection accuracy. Figure 1 shows the architectural layout of YOLOv8, which is segmented into three primary components:

1. Backbone: The backbone serves as a vital element in obtaining features from the input image. Primarily, the backbone of YOLOv8 constitutes CBS (Conv-BN-SiLU) and C2f modules, where CBS performs downsampling, while the C2f module is utilized for the extraction of features.
2. Neck: The neck component of YOLOv8 adopts the path aggregation network–feature pyramid network (PAN-FPN) structure, which introduces a bottom-up path based on an FPN. This path allows low-level features to be fused with high-level features again, helping to capture targets of varied dimensions and enhance the accuracy of object detection.
3. Head: The head section generates the final predictions, which encompass the positioning and dimensions of bounding boxes, along with the class probability for each box.

To address the limitations of anchor-based methods, YOLOv8 adopts an anchor-free prediction approach. This method directly predicts the object center, eliminating the need for predefined anchor boxes and reducing computational overhead by avoiding offset calculations. The anchor-free mechanism eliminates the need for pre-set anchor box dimensions, simplifying the model architecture and reducing the number of parameters. This enhances the model’s ability to detect objects of varying sizes more effectively. To improve the precision of bounding box regression, YOLOv8 incorporates the Distribution Focal Loss (DFL). The DFL is a specialized loss function for bounding box prediction that enhances localization accuracy by minimizing the discrepancy between the predicted and target edge position distributions. Let $t_{l}$ and $t_{r}$ denote the discretized distributions of the target box’s left and right edges, respectively, and $p_{l}$ and $p_{r}$ represent the corresponding predicted distributions. The length of the discretized distribution, denoted as *n*, indicates the number of possible discrete points for the edge positions of bounding boxes. The DFL computes the cross-entropy loss by comparing the predicted and target distributions for the positions of the left edge and the right edge, which are defined by

(1) $$L_{l}=-\sum_{i=0}^{n-1}t_{l}\left(i\right)log\left(p_{l}\left(i\right)\right)$$

and

(2) $$L_{r}=-\sum_{i=0}^{n-1}t_{r}\left(i\right)log\left(p_{r}\left(i\right)\right)$$

respectively, where $t_{l}\left(i\right)$ and $t_{r}\left(i\right)$ denote the probabilities at the *i*-th discretized position in the target distribution, $p_{l}\left(i\right)$ and $p_{r}\left(i\right)$ represent the corresponding probabilities in the predicted distribution. Note that DFL processes each edge of the bounding box independently. This allows the loss to be computed separately for each edge, with dynamic weight adjustments based on the relative positions of the predicted and target bounding boxes. This independent processing improves the localization accuracy of the bounding box, especially for asymmetric bounding boxes. In addition, by emphasizing regions with large discrepancies between predicted and target distributions, DFL effectively reduces positioning errors and enhances overall accuracy.

### 3.2. Overview of the RFAG-YOLO Architecture

**Figure 2:**
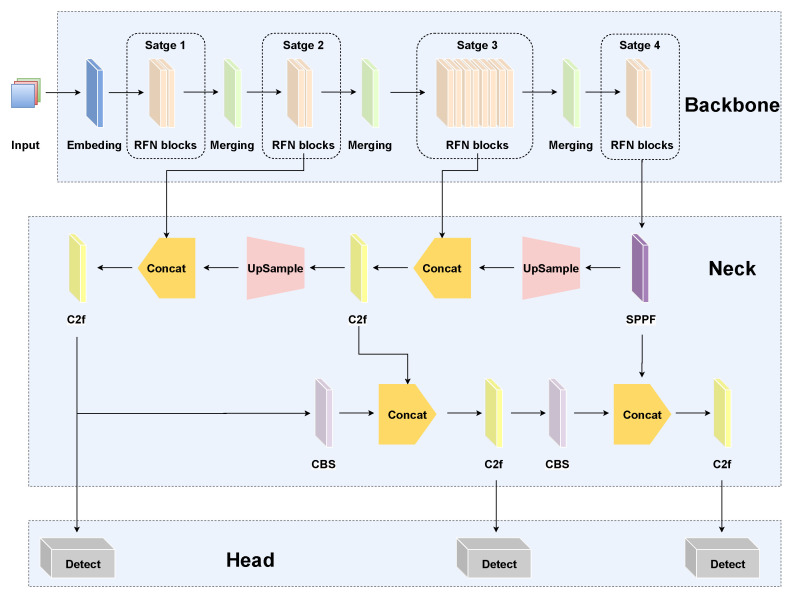
RFAG-YOLO network structure diagram.

Figure 2 illustrates the architectural design of the proposed RFAG-YOLO model, highlighting its key innovations aimed at improving object detection performance in UAV imagery. Compared to the baseline YOLOv8, the primary enhancements of RFAG-YOLO are concentrated in the core network architecture and the detection module, which are specifically designed to address the challenges of UAV-based object detection. Specifically, the backbone of RFAG-YOLO integrates FasterNet with the proposed RFN block, organized into a four-stage hierarchical architecture to optimize feature extraction at multiple scales. Each stage processes feature maps with downsampling ratios of 1/4, 1/8, 1/16, and 1/32, respectively, enabling the network to capture both fine-grained details and high-level semantic information across different scales. The neck of RFAG-YOLO incorporates YOLOv8’s PAN-FPN network, which combines a top-down FPN with bottom-up PANet to achieve efficient multi-level feature fusion, as well as the enhanced detection capability for targets at varying scales. In addition, this architecture employs iterative bidirectional feature transfer, effectively preserving high-resolution details while integrating deep semantic information. To address the limitations of single-scale feature maps, the SAF module, based on a scale attention mechanism, is integrated before the detection head. This module dynamically weights multi-scale features, enhancing the representation of fine-grained details and improving detection performance. Moreover, this integration allows RFAG-YOLO to precisely capture target object details during the prediction phase, leading to a significant improvement in localization accuracy and enhancing the model’s ability to understand complex scenes and detect objects at multiple scales.

### 3.3. The RFN Block

In high-altitude UAV images, small targets pose recognition challenges due to their limited pixel size and sparse feature details, which can be easily obscured by broad backgrounds such as terrain, buildings, and vegetation. As advancements are made in the fields of computer vision and deep learning, attention mechanisms have become a key strategy to address this challenge. In convolutional layers, the same kernel weights are applied across all spatial locations of the input feature map—this is known as the parameter sharing characteristic. While this characteristic helps reduce the total number of parameters and improves computational efficiency, it also means that the same feature extraction pattern is uniformly applied across the entire feature map. When using larger kernels, this uniform processing might not be optimal for small-object detection, as different regions of the feature map may require different processing patterns.

**Figure 3:**
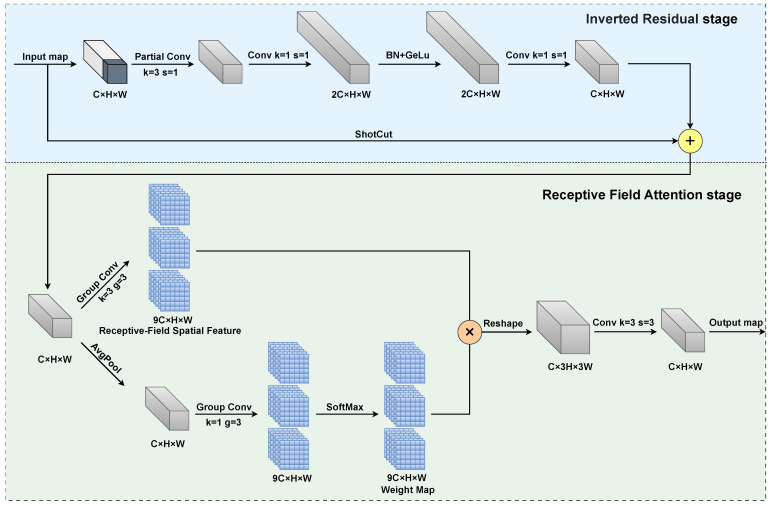
Structure of RFN block.

To surmount the aforementioned challenges, we propose an innovative feature extraction component, the RFN block, based on (receptive field attention) RFA. The architecture of the RFN block, as depicted in Figure 3, can be divided into two main stages. Initially, an inverted residual block, composed of a partial convolution coupled with two 1 × 1 convolutions, is employed to learn incremental information in relation to the original input. Subsequently, by adaptively modifying the weights at each position in the convolutional kernel through receptive field attention, the ability of the RFN block to capture key features in local regions is enhanced, effectively alleviating the parameter-sharing problem in traditional convolution.

As shown in Figure 3, in the residual structure stage, the main branch first employs a partial convolution layer with a kernel size of 3 × 3, which processes the first quarter of the feature map channels. This is followed by two point-wise convolution layers (1 × 1 convolutions). The output is then obtained through an element-wise addition of the main branch features with the input feature map. This efficient structure enables comprehensive channel-wise information utilization while maintaining low computational complexity.

The second stage of the RFN block is the receptive field attention module. For an input of dimensions C × H × W (channels, height, width), the RFA first employs a group convolution with a kernel size of 3 × 3 to expand the channel dimension, generating the receptive field space features. Secondly, through average pooling and a 1 × 1 group convolution, while preserving its spatial resolution, the feature map’s depth is enhanced. Then, softmax is used to normalize the positional weights within the feature map, resulting in a weight map. Here, the weight map can be seen as a set of convolution kernels. After performing a dot product operation at each position of the receptive field space feature map, we acquire a feature map that is weighted. Finally, the weighted feature map is reshaped into a shape of 3C × H × W, and the resolution is decreased using a standard 3 × 3 convolution, producing the final output.

In general, the formula for RFA is represented in Equation (3), where *F* is the input feature map, and *k* denotes the size of the convolution kernel. The output feature map $F^{\prime \prime }$ is obtained by multiplying element-by-element the attention feature map *A* and the transformed receptive field space feature map $F^{\prime }$.

(3) $$\begin{array}{cc} F^{\prime \prime } & =Softmax\left(GroupConv^{\left(1\times 1\right)}\left(AvgPool\left(F\right)\right)\right)\times ReLu\left(Norm\left(GroupConv^{\left(k\times k\right)}\left(F\right)\right)\right)\\ \; & =A\times F^{\prime } \end{array}$$

### 3.4. Improved FasterNet Backbone Based on RFN Block

Backbone networks are generally used for feature extraction of input images. Small targets in high-resolution feature maps contain detailed spatial information, which is crucial for accurate detection. However, through the downsampling process, while the receptive field increases and semantic information is enriched, the fine-grained features of small objects may become less distinct. To address this challenge, we introduce an improved FasterNet based on RFN blocks as the backbone network of RFAG-YOLO. This architecture combines the segmented structure of FasterNet with the efficient feature extraction capability of RFN blocks, thus enhancing the sensitivity of the model in identifying small targets.

**Figure 4:**
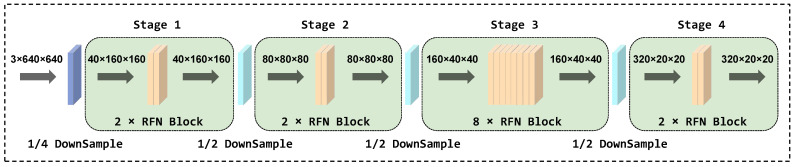
Structure of improved FasterNet.

As shown in Figure 4, the improved FasterNet consists of four consecutive stages, each stage starting with an embedding or merging layer and equipped with multiple RFN blocks. The embedding layer uses a 4 × 4 standard convolution with a stride of 4, while the merging layer uses a 2 × 2 standard convolution with a stride of 2. These embedding or merging layers have two main functions: first, to decrease the spatial scales of the input, thereby effectively reducing the image resolution; the second is to increase the channel capacity to improve the model’s representation capability. In the first and second stages, we deployed two RFN blocks, respectively. For initial feature extraction, it is crucial to preserve as many original image details as possible, especially when handling with small targets. In the third stage, we incorporated eight RFN blocks. At this point, the image resolution is lower, but the network gains a more abstract and comprehensive understanding of the image’s content. This stage facilitates identifying detailed features, aiding in the detection of small objects in various backgrounds. Finally, in the fourth stage, the number of RFN blocks is reduced back to two. This stage aims to refine the features extracted in the previous stages and prepare them for the final prediction. Additionally, the reduction in the number of building blocks also aids in managing the model’s complexity and lowering its computation demands.

The integration of RFN blocks with FasterNet offers several key advantages, enhancing the model’s capability to address the challenges of small-object detection in complex scenes. First, the residual structure of the RFN block, combined with the receptive field attention mechanism, significantly enhances FasterNet’s feature extraction efficiency. This improvement enables the model to better capture the contextual information of small targets, which is critical for accurate detection in cluttered environments. Second, to account for the varying resolutions of feature maps at different stages, a hierarchical arrangement of RFN blocks is implemented within FasterNet. This hierarchical arrangement ensures that each stage effectively utilizes the unique characteristics of its feature maps, leading to effective improvements in the overall detection accuracy and robustness. Furthermore, by merging the channel extension and spatial dimension compression of RFN blocks, the representation ability of the model is effectively improved. Finally, the integration of RFN blocks with FasterNet not only improves the effectiveness of small-target detection, but also maintains a high inference speed, making it suitable for real-time applications.

### 3.5. Feature Fusion Module Based on Scale Attention

The YOLOv8 detection head plays a critical role in the object detection pipeline, producing final detection results by processing multi-scale feature maps extracted by the FPN. The detection head processes feature maps at three distinct scales, with spatial resolutions of 80 × 80, 40 × 40, and 20 × 20, corresponding to the detection of small objects (larger than 8 × 8 pixels), medium objects (larger than 16 × 16 pixels), and large objects (larger than 32 × 32 pixels), respectively. This multi-scale design enables the model to effectively handle objects of varying sizes. However, this multi-scale detection approach encounters two significant challenges that can degrade detection performance. First, low-resolution feature maps often fail to capture fine-grained details of small objects, resulting in inaccurate localization and reduced detection confidence. Second, in high-resolution feature maps, the dominant feature responses of large objects can suppress the weaker responses of small objects, particularly due to the spatial influence of large objects on neighboring regions. This phenomenon significantly compromises the detection and classification accuracy of small objects. To mitigate these challenges, we propose a SAF module, which is integrated before the detection head of RFAG-YOLO to enhance multi-scale feature representation. The SAF module facilitates adaptive feature processing across different scales by dynamically weighting and integrating information from all three resolution levels. This approach significantly improves the model’s ability to localize and classify objects, particularly small objects, with higher accuracy.

**Figure 5:**
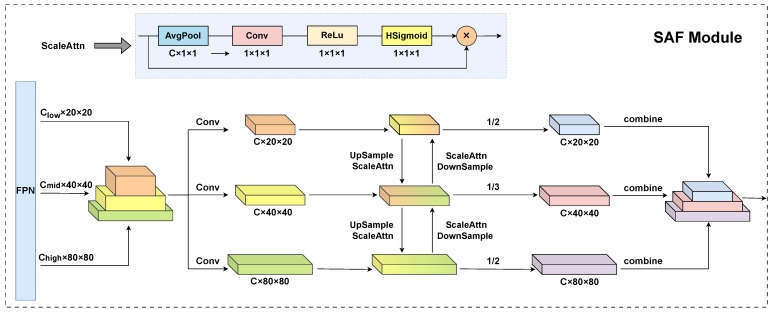
Structure of SAF module.

The scale attention module generates attention weights through a sequence of carefully designed operations, as illustrated in Figure 5. This module aims to dynamically adjust feature representations based on their scale relevance, enhancing the model’s ability to handle multi-scale objects. First, adaptive average pooling is employed to aggregate spatial information across the width and height dimensions of the feature map, effectively summarizing global contextual information while reducing computational complexity. This operation reduces the spatial dimensions to C × 1 × 1, capturing global contextual information from the entire feature map and enabling efficient computation of attention weights. Next, a 1 × 1 convolutional layer is utilized to process the pooled features, reducing the channel dimension and facilitating the extraction of scale-relevant information. This layer reduces the channel dimension to 1, compressing the multi-channel information into a single-channel representation that encapsulates the most salient scale-related features. The compressed features are then passed through a ReLU activation function, introducing non-linearity to enhance the expressiveness of the attention mechanism and improve its ability to model complex relationships. Finally, an HSigmoid activation function is applied to normalize the attention weights to the range [0,1], ensuring that the weights are suitable for adaptive feature scaling and enhancing the stability of the attention mechanism. The generated weights are then used to adaptively modulate the corresponding feature maps, enabling a scale-aware feature refinement that enhances the model’s ability to detect objects across varying scales with a higher accuracy.

As illustrated in Figure 5, the SAF module receives feature maps from three different levels of the FPN. Initially, we employ convolutions to adjust the channel dimensions of all feature maps to a consistent size. Subsequently, a spatial resolution alignment is achieved through upsampling or downsampling operations on these feature maps. For the multi-scale feature fusion process, we define the following notations, where $F_{i}$ denotes the feature map at the *i*-th level, $F_{i}^{\prime }$ denotes the fused feature map at the *i*-th level, ScaleAttn(·) represents the scale attention function, down(·) and up(·) denotes the downsampling and upsampling operations, respectively.

- For the feature map with the lowest resolution (20 × 20), SAF first downsamples the feature map from the higher level to match its resolution. Subsequently, scale attention is utilized on the downsampled feature map for weighting. The weighted feature map is then added to the current feature map and divided by 2, resulting in the fused feature map for this level, thereby adding it to the output list. This step can be represented by
  (4) $$F_{low}^{\prime }=\frac{F_{low}+ScaleAttn\left(down\left(F_{mid}\right)\right)}{2}$$
- For the feature map with the highest resolution (80 × 80), SAF uses bilinear interpolation to upsample the feature map from the lower level to match its resolution. Then, similar to the process for the lowest resolution, scale attention is applied for weighting. The weighted feature map is combined with the current feature map, and the result is divided by 2, yielding the fused feature map for this level; it is subsequently added to the output list and can be represented by
  (5) $$F_{high}^{\prime }=\frac{F_{high}+ScaleAttn\left(up\left(F_{mid}\right)\right)}{2}$$
- For the intermediate-level feature map (40 × 40), SAF applies weighting and fusion to both the upper and lower level feature maps, dividing the result by 3 to obtain the fused feature map for this level, and then accordingly adding it to the output list. This step is represented by
  (6) $$F_{mid}^{\prime }=\frac{F_{mid}+ScaleAttn\left(down\left(F_{high}\right)\right)+ScaleAttn\left(up\left(F_{low}\right)\right)}{3}$$

## 4. Experiment

This section presents a comprehensive experimental evaluation of the proposed RFAG-YOLO model, aiming to validate its effectiveness in addressing the challenges of UAV-based object detection, such as small object sizes and complex backgrounds. We first compared our model with baseline YOLOv8 on the VisDrone2019 [44] dataset, demonstrating significant improvements in small-object detection while maintaining computational efficiency. Next, ablation studies were conducted to systematically evaluate the contributions of each proposed component, including the enhanced FasterNet backbone, RFN blocks, and SAF module. Comparisons with state-of-the-art detection models further confirmed RFAG-YOLO’s superior performance. Finally, to further analyze the model’s behavior, visualization experiments were conducted using confusion matrices and Grad-CAM. These experiments revealed that RFAG-YOLO significantly improved the localization and classification accuracy of small objects, even in complex drone-captured scenes with occlusions and cluttered backgrounds.

### 4.1. Dataset

**Figure 6:**
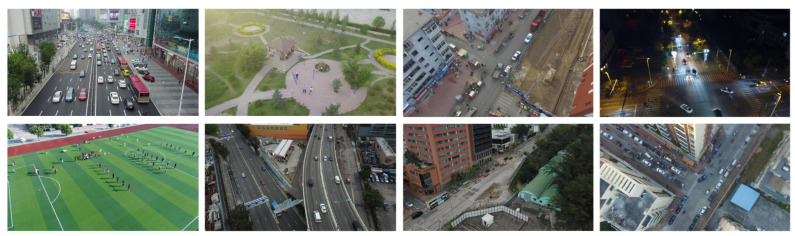
Some of the images in the VisDrone2019 dataset.

Our proposed model was rigorously evaluated on the VisDrone2019 aerial image dataset, a comprehensive and diverse collection of images curated by the AISKYEYE team from the Machine Learning and Data Mining Laboratory at Tianjin University, China. This dataset is specifically designed for UAV vision applications, offering a rich variety of scenarios to test the robustness and generalizability of object detection models. The dataset was split into training, validation, and testing subsets, with 6471, 548, and 1610 images in each subset, respectively. Representative examples from the VisDrone2019 dataset are illustrated in Figure 6, showcasing the diversity of scenarios, as well as the challenges posed by varying lighting conditions, object scales, and background complexity. The dataset encompasses a wide range of real-life scenarios, including urban, rural, and highway environments, with 10 distinct object categories such as pedestrians, bicycles, cars, vans, buses, and motorcycles. This diversity ensures that the dataset is well-suited for evaluating the robustness and generalizability of object detection models in real-world UAV applications.

The VisDrone2019 dataset includes a wide variety of challenging scenarios that accurately reflect real-world operational conditions, making it an invaluable resource for evaluating object detection algorithms. This dataset includes densely crowded scenes characterized by significant object overlaps and interactions, such as busy intersections and parking lots with high concentrations of vehicles and pedestrians. These scenarios pose significant challenges for object detection due to occlusions and spatial ambiguities. The VisDrone2019 dataset features a wide variety of weather conditions, including clear skies, rain, and fog, as well as diverse lighting conditions such as bright daylight, low-light evening scenarios, and strong shadows. These variations can test the robustness of detection algorithms under different environmental conditions. Additionally, the images in the VisDrone2019 dataset contain varying levels of occlusion, where objects are partially obscured by structures, vegetation, or other objects, as well as diverse viewing perspectives resulting from different UAV flight altitudes and camera angles. These factors further complicate the detection task, making the VisDrone2019 dataset an exemplary benchmark for rigorously evaluating the performance and robustness of object detection approaches under realistic and varied operational conditions.

**Figure 7:**
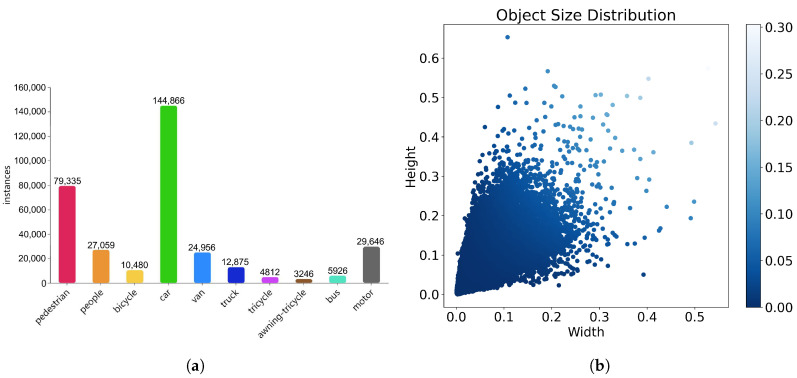
Statistical analysis of category and target size distributions in the VisDrone2019 dataset. (**a**) Distribution of categories in the VisDrone2019 dataset. (**b**) Distribution of target sizes in the VisDrone2019 dataset.

Figure 7 shows some information about the VisDrone2019 dataset. Panel (a) shows the distribution of various object labels in the dataset. Pedestrians and vehicles dominate the annotations, while the remaining categories account for a smaller portion. Panel (b) shows a two-dimensional scatter plot that reveals the aspect ratio distribution of objects in the image relative to their height and width. The figure shows a darker color concentration in the lower left, indicating that smaller objects dominate in the dataset. This visual analysis highlights that the dataset emphasizes the detection of small targets.

### 4.2. Evaluation Metrics

In object detection tasks, the following terminology and metrics are commonly employed to evaluate the relationship between the model’s predictions and the ground truth labels, providing a foundation for performance assessment and algorithm optimization.

1. Intersection over Union (IoU): IoU is a fundamental metric in object detection, quantifying the overlap between predicted and ground truth bounding boxes. It plays a pivotal role in evaluating detection accuracy and is integral to the NMS process, where it determines the redundancy of overlapping predictions. Mathematically, IoU can be defined as
  (7) $$IoU=\frac{OverlapArea}{UnionArea}=\frac{A_{int}}{A_{pred}+A_{act}-A_{int}}$$
  where $A_{int}$ denotes the area of intersection between the predicted bounding box and the ground truth bounding box; $A_{pred}$ and $A_{act}$ represent the areas of the predicted bounding box and the ground truth bounding box, respectively.
2. NMS: In object detection tasks, models frequently generate multiple overlapping bounding boxes for a single object due to the sliding window or anchor-based detection mechanisms. NMS is employed to eliminate redundant predictions by first sorting the bounding boxes based on their confidence scores. The box with the highest confidence is selected as the final detection, while all other boxes with an IoU exceeding a predefined threshold are suppressed. This process is iteratively applied to all remaining boxes, ensuring a precise and non-redundant set of detection results.
3. True Positive (TP): This metric indicates that the model correctly detects a target, as determined by an IoU between the predicted bounding box and the ground truth bounding box exceeding a predefined threshold. This metric is essential for evaluating the model’s detection accuracy.
4. False Positive (FP): An FP occurs when the model incorrectly detects a nonexistent target or misclassifies background regions as a specific object category. This type of error, often termed a false alarm, can significantly impact the model’s precision and overall reliability.
5. False Negative (FN): An FN occurs when the model fails to detect an actual target present in the image. This type of error, commonly referred to as a miss, can reduce the model’s recall and is often caused by small object sizes, occlusions, or complex backgrounds.

Building on these fundamental concepts, several evaluation metrics are employed in this study to comprehensively assess the performance of the proposed model.

1. Precision: Precision quantifies the proportion of true positive (TP) detections relative to the total number of detections (TP + FP), as defined by
  (8) $$Precision=\frac{TP}{TP+FP}$$
  Precision is a critical metric for evaluating the model’s ability to minimize false positives, particularly in scenarios where false alarms are costly.
2. Recall: Recall measures the fraction of true positive (TP) detections relative to the total number of actual objects (TP + FN), as defined by
  (9) $$Recall=\frac{TP}{TP+FN}$$
  This metric evaluates the model’s ability to identify all relevant targets, particularly in scenarios where missing detections is undesirable. A high recall value indicates a low false negative rate, reflecting the model’s robustness in detecting true targets.
3. Mean Average Precision (mAP): mAP is a comprehensive metric that integrates precision and recall by calculating the average precision (AP) at various recall levels. It provides an overall evaluation of model performance across all object categories, making it a key metric in object detection research. To compute mAP, the AP for each category is first determined by calculating the area under the precision–recall curve. These AP values are then averaged across all categories. Mathematically, mAP can be defined by
  (10) $$mAP=\frac{1}{N}\sum_{i=1}^{n}AP_{i}$$
  where $AP_{i}$ denotes the average precision for the *i*-th category, and *N* represents the total number of categories.

### 4.3. Experimental Environment

In this study, the training, validation, and testing phases were conducted under identical experimental conditions to ensure consistency and reproducibility. Notably, all training processes were performed from scratch without the use of pretrained weights, allowing for a fair evaluation of the model’s learning capabilities. The detailed hardware configuration and hyperparameter settings are summarized in Table 1 and Table 2, respectively.

### 4.4. Comparison of RFAG-YOLO with Baseline Model YOLOv8

To validate the performance of RFAG-YOLO in identifying small objects within drone-captured imagery, we performed comparative experiments using the widely recognized VisDrone2019 public dataset. In these experiments, we maintained consistency with other training conditions to compare the performances of RFAG-YOLO, YOLOv8n, and YOLOv8s.

**Figure 8:**
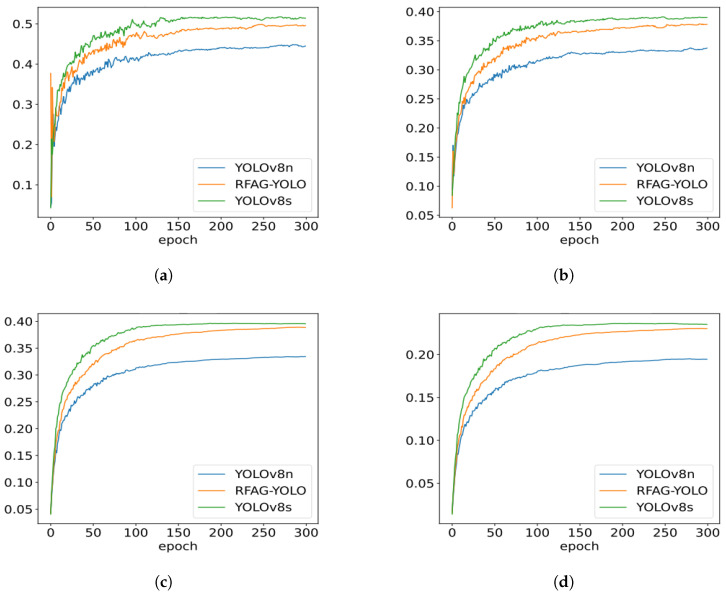
Training curves comparison of YOLOv8n, RFAG-YOLO, and YOLOv8s on the VisDrone2019 dataset. (**a**) Precision curves during training. (**b**) Recall curves during training. (**c**) mAP50 curves during training. (**d**) mAP50-95 curves during training.

Figure 8 shows the performance trends of YOLOv8n, RFAG-YOLO, and YOLOv8s during training. It can be observed that throughout the training process, the performance of the RFAG-YOLO model consistently surpasses that of YOLOv8n, and its performance curve is closer to that of YOLOv8s. This indicates that RFAG-YOLO not only performs well in terms of the final performance but also exhibits higher stability during training. Additionally, as the number of training epochs increases, the performance improvement of RFAG-YOLO becomes more significant, ultimately reaching a higher accuracy level, which suggests that the model has a stronger ability to learn from complex scenarios.

Table 3 provides specific performance metrics for YOLOv8n, RFAG-YOLO, and YOLOv8s. According to the data, the RFAG-YOLO model achieved a detection accuracy of 49.6%, a significant improvement over YOLOv8n (44.5%). Simultaneously, the recall rate of RFAG-YOLO also increased, reaching 37.8%, which is higher than that of YOLOv8n, which is 33.8%. Furthermore, the mAP50 indicator of RFAG-YOLO reached 38.9%, very close to the level of YOLOv8s. Importantly, under stringent conditions requiring a high confidence, the mAP50-95 indicator of RFAG-YOLO reached 23.1%, significantly superior to YOLOv8n (19.5%). These data underscore that RFAG-YOLO maintains high stability and accuracy under various confidence thresholds.

In terms of resource efficiency, RFAG-YOLO requires more parameters (5.94 M) and incurs a higher computational cost (15.7 GFLOPs) than YOLOv8n, but compared with YOLOv8s, these increases are relatively balanced, considering the substantial improvement in detection performance. We believe this is a wise trade-off between model complexity and accuracy. In addition, despite the relatively low frames per second (FPS) rate compared to YOLOv8n and YOLOv8s, RFAG-YOLO still maintains sufficient real-time processing capabilities to meet the needs of applications such as real-time object detection in drone images.

From Table 4, RFAG-YOLO consistently outperforms YOLOv8n in terms of the mAP50 indicator across all categories. Notably, the performance boost is particularly pronounced for structurally intricate or small-scale object categories such as “bicycles”, “tricycles”, and “awning-tricycles”, underscoring its capability to handle complex scenes and enhance the detection of minute details. Furthermore, RFAG-YOLO surpasses even the larger YOLOv8s model in terms of detection precision for categories such as “truck”, demonstrating that our improvements go beyond simple model scaling.

In summary, through meticulously designed architectural refinements, RFAG-YOLO achieves the dual optimization of detection precision and efficiency, striking a favorable balance between computational resources and performance enhancement. It delivers excellent precision, recall, and mAP metrics and optimizes computational resource utilization. Therefore, it offers an efficient and practical approach for real-world applications, especially in scenarios requiring high-accuracy object detection.

### 4.5. Ablation Experiments

The unmodified YOLOv8n served as the baseline model, and we performed a series of ablation experiments on the VisDrone2019 dataset to validate the efficacy of our proposed improvements. Initially, we replaced the original backbone of YOLOv8n with FasterNet and evaluated the performance changes. Subsequently, we gradually integrated other innovative techniques, such as the SAF model and RFN block, to further improve model performance. Table 5 summarizes the results of our extensive ablation experiments, where the activation of a specific module is denoted by ✓ and the disabling of a component is indicated by the symbol ✗. The table presents the incremental contributions of each component to the overall performance improvement.

In the ablation experiments, we systematically assessed the individual contributions of key components in the RFAG-YOLO model, using the VisDrone2019 validation set as our benchmark. Our findings, as presented in Table 5, reveal that the baseline YOLOv8n achieved a precision of 44.5% and an mAP50 of 33.5%. On introducing the FasterNet backbone, the model accuracy rose to 46.5%, and its mAP50 reached 35.3%. This significant improvement can be attributed to FasterNet’s design for efficient operations. Its multistage architecture naturally yields feature maps of varying scales—this capability is vital for object detection tasks in which targets of different sizes may be best detected at distinct feature levels.

Subsequently, the introduction of the SAF module, even without FasterNet, led to noticeable increments in both the recall and mAP metrics. This result proves that the mechanism improves the feature representation across varying scales. The SAF module’s effectiveness stems from its ability to perform weighted fusion of multi-scale feature maps before the detection head, enriching the detailed information at each scale and improving the model’s feature representation capabilities.

When FasterNet was combined with the RFN block (without SAF module), a new threshold of performance was reached with an accuracy of 49.2% and an mAP50 of 37.2%. This substantial improvement can be explained by the RFN block’s unique design: while traditional convolutional layers struggle to accurately capture intricate local features, our RFN block incorporates learnable weight maps that adapt convolution kernel weights based on the input image features. This distinctive capability enables the network to concentrate on critical areas while disregarding irrelevant background details, thus enhancing the model’s performance in detecting small targets.

The distribution of RFN blocks across the FasterNet stages was carefully optimized to balance computational efficiency and feature expression. By varying the number of RFN blocks across the stages in accordance with the feature map scales and complexities, we achieved an adaptive optimization of receptive fields. This ensured an optimal feature expression across all levels, from fine-grained details to broader semantic understandings. To maintain computational efficiency, we deployed fewer blocks in early stages to minimize expensive computations on high-resolution feature maps, while increasing block numbers in deeper stages where feature maps are smaller, efficiently boosting high-level semantic feature extraction.

Ultimately, the RFAG-YOLO model exhibited its best performance when FasterNet, RFN block, and the SAF module were applied together—with the accuracy, recall, and mAP50 increasing impressively to 49.6%, 37.8%, and 38.9%. These outcomes underscore not only the individual effectiveness of each technology but also their synergistic interplay, jointly propelling a substantial leap in model performance.

### 4.6. Comparison with Other Advanced Models

To demonstrate the advantages of RFAG-YOLO compared to other leading object detection models, we performed a comprehensive comparative analysis against various state-of-the-art detection approaches. The comparison included CNN-based models such as YOLOv5n, YOLOv7, YOLOv8n, TPH-YOLO, YOLOv10n, and YOLOv11n, as well as transformer-based architectures including RT-DETR-R18 and D-FINE-S. All models were evaluated under identical training conditions to ensure a fair comparison.

As seen from Table 6, RFAG-YOLO demonstrates impressive detection capabilities with a good balance between accuracy and computational efficiency. While RT-DETR-R18 achieves slightly higher mAP metrics (mAP50 of 42.5% and mAP50-95 of 24.5%), it requires significantly more computational resources with 19.9M parameters and 57.0 G FLOPs. In contrast, RFAG-YOLO achieves competitive performance (mAP50 of 38.9% and mAP50-95 of 23.1%) with only 5.9M parameters and 15.7G FLOPs, showing superior efficiency. Compared to TPH-YOLO, a model customized for drone image detection, RFAG-YOLO achieves notable enhancements in mAP50 (by 6.0%) and mAP50-95 (by 5.4%). Furthermore, compared with the latest version of the YOLO model, i.e., YOLOv11, RFAG-YOLO demonstrates superior performance, with improvements of 5.4% and 3.6% in mAP50 and mAP50-95, respectively.

In addition, as shown in Table 7, RFAG-YOLO exhibits exceptional performance across most categories in the VisDrone2019 dataset. Particularly in crucial categories such as pedestrians and cars, our model achieves impressive mAP50 scores of 41.4% and 79.3%, respectively, significantly outperforming most lightweight models. While RT-DETR-R18 and D-FINE-S show slightly higher scores in some categories, they come at the cost of a much higher computational complexity. RFAG-YOLO maintains a strong balance between accuracy and efficiency, demonstrating its practical value in real-world drone detection applications.

### 4.7. Interpretability Experiments

We conducted a detailed analysis of the performance and characteristics of RFAG-YOLO using confusion matrices, class activation map visualizations, and final detection result plots. This comprehensive method offered insights into the model’s recognition accuracy and generalization capabilities, while also uncovering its internal decision-making mechanisms.

**Figure 9:**
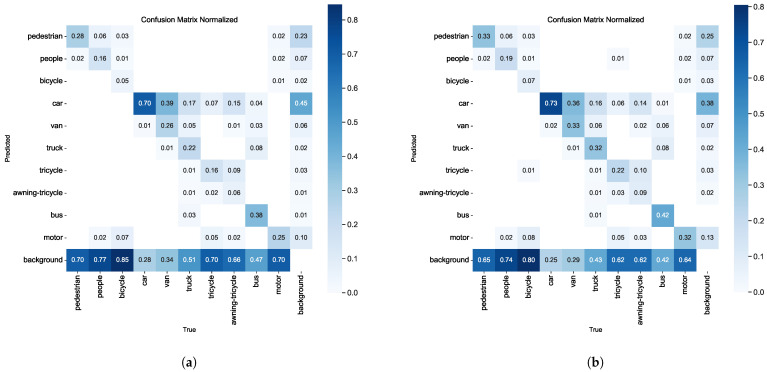
Normalized confusion matrices comparison between different models on the VisDrone2019 dataset. (**a**) Confusion matrix of YOLOv8n model. (**b**) Confusion matrix of our proposed RFAG-YOLO model.

The confusion matrix analysis, as depicted in Figure 9, reveals significant improvements in classification accuracy. The RFAG-YOLO confusion matrix shows higher values on its main diagonal compared to the YOLOv8n confusion matrix, indicating a higher probability of correct classification. This improvement can be attributed to RFA’s ability to generate receptive field space feature maps according to kernel sizes through grouped convolutions and global average pooling, thus preserving the spatial structure while enhancing channel independence. Despite the introduction of additional computational steps for feature refinement, RFA maintains high computational efficiency through designs such as 1 × 1 convolutions and grouping strategies.

Additionally, compared with YOLOv8n, RFAG-YOLO has a lower value in the lower left triangle region, indicating a lower probability of missed detections. This improvement demonstrates that RFAG-YOLO can better capture all relevant objects in the scene, effectively addressing the insufficient detection problems of its predecessor.

**Figure 10:**
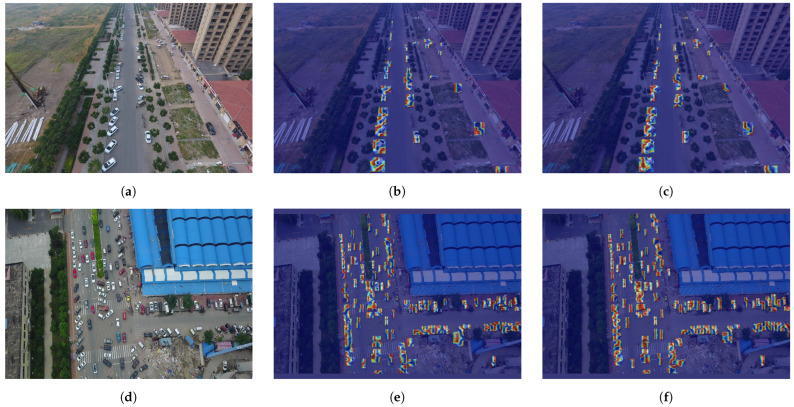
Comparison of detection heatmap visualization on the VisDrone2019 dataset. (**a**,**d**) Original drone-captured images showing complex parking scenarios. (**b**,**e**) Heatmap visualization of YOLOv8n detection outputs. (**c**,**f**) Heatmap visualization of our proposed RFAG-YOLO detection outputs.

Our Grad-CAM analysis provides further insights into the model’s attention mechanisms. As shown in Figure 10, the RFAG-YOLO heatmaps demonstrate more focused and precise patterns of high activation zones, even for tiny targets that are usually overlooked. These visualizations highlight how our architectural improvements, particularly the RFN block and SAF module, work together to enhance the model’s sensitivity to fine details and edges. The warmer colors in critical regions indicate that our model successfully learns to allocate attention to relevant features while suppressing background noise.

**Figure 11:**
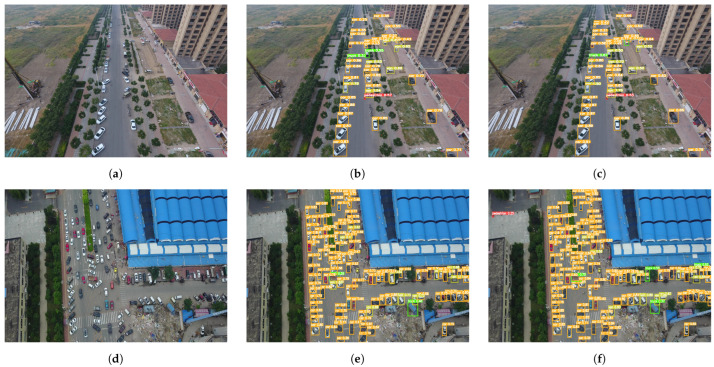
Comparison of object detection results on the VisDrone2019 dataset. (**a**,**d**) Original drone-captured images showing complex parking scenarios with multiple vehicles. (**b**,**e**) Detection results obtained by YOLOv8n. (**c**,**f**) Detection results obtained by our proposed RFAG-YOLO model.

The qualitative detection results illustrated in Figure 11 provide a compelling empirical validation of our theoretical improvements. Under identical testing conditions, RFAG-YOLO demonstrates superior localization precision for small-scale objects, exhibiting both reduced false positives and significantly fewer missed detections compared to the baseline model. This enhanced detection capability is particularly evident in challenging scenarios with dense object distributions and complex backgrounds. The robust performance across objects of varying scales can be attributed to the synergistic effect of our architectural innovations: FasterNet’s intrinsic multi-scale feature hierarchy effectively captures objects at different resolutions, while the SAF module’s adaptive feature fusion mechanism intelligently aggregates and refines these multi-scale representations. These qualitative results align with our quantitative findings and further validate the effectiveness of our proposed improvements in real-world detection scenarios.

## 5. Discussion

Our proposed RFAG-YOLO model demonstrated significant improvements over the baseline YOLOv8n model on the VisDrone2019 dataset, particularly in small-object detection tasks. This success can be attributed to the RFN module’s learnable weight maps, which adaptively adjust convolution kernel weights based on input image features. Building upon the insights from Zhang et al. [16], who revealed the limitations of traditional spatial attention in addressing convolutional kernel parameter sharing, our RFN module leverages receptive field attention (RFA) to focus on both receptive-field spatial features and effective attention weights for large-size convolutional kernels, offering significant performance improvements with minimal computational overhead. Furthermore, our SAF module implements scale attention mechanisms for weighted fusion of multi-scale feature maps, enhancing the model’s feature representation capabilities while maintaining computational efficiency through grouped convolutions and strategic pooling operations.

While recent transformer-based detection models like RT-DETR and D-FINE have demonstrated remarkable accuracy through their end-to-end architecture and innovative bounding box regression, our experimental analysis reveals their limitations in UAV applications. The computational overhead of self-attention mechanisms and complex architectures poses significant challenges for deployment on resource-constrained UAV platforms. Inspired by the efficient design principles of FasterNet [15], RFAG-YOLO adopts a stage-wise architecture that strategically distributes RFN blocks across different network levels. This approach achieves an optimal balance between detection performance and computational efficiency. The adaptive allocation of computational resources across scales enables effective feature extraction while maintaining efficiency, making RFAG-YOLO particularly well-suited for real-world UAV applications.

Despite the promising results, our approach has several limitations that warrant discussion. The RFN block, while effectively enhancing feature representation, introduces additional parameters and slight computational overhead compared to standard convolutions. Moreover, although our SAF module improves multi-scale feature fusion, its effectiveness may be constrained by the fixed input resolution of 640 × 640 pixels, which could limit the detection of extremely small objects in high-resolution UAV imagery.

Looking ahead, we identify two key directions for future research. First, we aim to explore more lightweight attention mechanisms to further reduce computational costs while maintaining detection accuracy, potentially drawing inspiration from efficient design principles of recent transformer-based models. Second, considering that modern high-speed cameras now offer several megapixels of resolution at high frame rates, we plan to investigate methods to process higher-resolution input images beyond the current 640 × 640 input size and optimize the subsequent downsampling strategies in feature extraction, as this limitation may fundamentally affect small-object detection performance. These enhancements will further strengthen RFAG-YOLO’s potential for practical UAV applications.

## 6. Conclusions

In this paper, we propose RFAG-YOLO, an advanced object detection framework specifically designed to address the challenges of small-object detection in UAV imagery. By integrating the novel RFN block and an enhanced FasterNet backbone, RFAG-YOLO significantly improves the model’s ability to capture fine-grained local details and leverage scale features. The introduction of the SAF module further enhances the model’s robustness by dynamically weighting features from different layers, enabling richer information flow and improved detection accuracy under complex conditions. Extensive experiments on the VisDrone2019 dataset demonstrated the superiority of RFAG-YOLO over state-of-the-art models, including YOLOv7, YOLOv8, YOLOv10, and YOLOv11. Specifically, RFAG-YOLO achieved an mAP50 of 38.9%, representing substantial improvements of 12.43% over YOLOv7, 5.99% over YOLOv10, and 16.12% over YOLOv8n and YOLOv11. Furthermore, RFAG-YOLO achieved 97.98% of the mAP50 performance of the larger YOLOv8s model while utilizing only 53.51% of its parameters, highlighting its exceptional efficiency in terms of the performance-to-parameter ratio. These results underscore the model’s suitability for resource-constrained UAV applications, where computational efficiency and detection accuracy are critical. The success of RFAG-YOLO lies in its ability to balance semantic information across multi-resolution feature maps, effectively address the limitations of traditional convolutional layers, and dynamically adapt to scale variations and complex backgrounds. These advancements make RFAG-YOLO a promising solution for real-world UAV applications, particularly in scenarios requiring an accurate detection of small objects under challenging conditions such as varying lighting, complex backgrounds, and diverse scales.

## Figures and Tables

**Table 1:** Hardware and software configuration details.

Parameters	Configuration
CPU	INTEL (Santa Clara, CA, USA) i7-9700k
GPU	NVIDIA (Santa Clara, CA, USA) RTX 2060
RAM	32 GB
GPU Memory	6 GB
Operating System	Ubuntu22.04
Programming Language and Libraries	Python (3.8.16)
CUDA	12.0
Pytorch	1.13.1

**Table 2:** Hyperparameters for training settings.

Hyperparameters	Value
Input Image Size	640 × 640
Number of Epochs	300
Batch Size	8
Optimizer	SGD
Learning Rate	0.01

**Table 3:** Comparison of RFAG-YOLO with YOLOv8n and YOLOv8s on validation data from the VisDrone2019 dataset.

Model	Precision (%)	Recall (%)	mAP50 (%)	mAP50-95 (%)	Params (M)	GFLOPs (G)	FPS
YOLOv8n	44.5	33.8	33.5	19.5	3.2	8.1	121.1
RFAG-YOLO	49.6	37.8	38.9	23.1	5.94	15.7	82.0
YOLOv8s	51.6	38.8	39.7	23.8	11.1	28.5	116.0

**Table 4:** Comparison of mAP50 for each category on the VisDrone2019 dataset.

Category	YOLOv8n	RFAG-YOLO	YOLOv8s
pedestrian	35.1	41.4	43.0
people	26.7	31.3	33.2
bicycle	8.2	11.1	12.8
car	76.1	79.3	79.8
van	39.0	44.1	44.7
truck	30.0	37.6	36.5
tricycle	22.4	28.8	28.0
awning-tricycle	11.6	16.7	15.9
bus	48.5	55.6	57.0
motor	36.7	43.0	45.3

**Table 5:** Results of ablation experiments on the VisDrone2019 dataset, where bold red font indicates the best performance.

FasterNet	RFN Block	SAF Module	Precision (%)	Recall (%)	mAP50 (%)	mAP50-95 (%)
✗	✗	✗	44.5	33.8	33.5	19.5
✓	✗	✗	46.5	34.4	35.3	20.6
✗	✗	✓	46.9	35.1	35.9	21.3
✓	✓	✗	49.2	36	37.2	21.9
✓	✓	✓	**49.6**	**37.8**	**38.9**	**23.1**

**Table 6:** Comparison of RFAG-YOLO with other models on the VisDrone2019 dataset.

Model	mAP50 (%)	mAP50-95 (%)	Params (M)	GFLOPs (G)
YOLOv5n	32.9	19.0	2.6	7.7
YOLOv7	34.6	18.0	6.2	13.8
YOLOv8n	33.5	19.5	3.2	8.1
TPH-YOLO	32.9	17.7	7.2	36.8
YOLOv10n	36.7	19.6	2.3	6.7
YOLOv11n	33.5	19.5	2.6	6.5
RFAG-YOLO	38.9	23.1	5.9	15.7
D-FINE-S	42.3	23.4	10.2	24.9
RT-DETR-R18	42.5	24.5	19.9	57.0

**Table 7:** Comparison of the average precision across categories of the VisDrone2019 dataset.

Category	YOLOv5n	YOLOv7	YOLOv8n	TPH-YOLO	YOLOv10n	YOLOv11n	RFAG-YOLO	D-FINE-S	RT-DETR-R18
pedestrian	34.6	41.2	35.1	41.1	34.7	35.4	41.4	43.5	44.9
people	27.6	37.0	26.7	32.9	27.8	27.7	31.3	39.0	39.2
bicycle	8.3	7.7	8.2	9.9	8.0	8.2	11.1	20.5	18.8
car	75.4	77.5	76.1	73.7	75.4	76.1	79.3	81.0	81.7
van	38.3	36.7	39.0	35.2	37.3	39.9	44.1	47.2	48.3
truck	28.6	28.1	30.0	27.3	28.7	28.8	37.6	36.2	36.2
tricycle	22.0	18.8	22.4	18.4	19.9	21.1	28.8	31.5	32.0
awning-tricycle	12.1	9.7	11.6	10.3	11.6	12.3	16.7	17.5	15.9
bus	46.5	44.9	48.5	41.4	45.8	48.6	55.6	53.8	54.9
motor	35.5	44.5	36.7	38.8	36.7	37.1	43.0	54.5	53.5

## Data Availability

Data are contained within the article.

## Abbreviations

The following abbreviations are used in this manuscript:

YOLO	You Only Look Once
RFAG-YOLO	receptive field attention-guided YOLO
RFN	receptive field network
SAF	scale-aware feature
RPN	Region Proposal Network
SSD	Single Shot MultiBox Detector
PGI	Programmable Gradient Information
NMS	Non-Maximum Suppression
SAS-FPN	multi-scale feature pyramid network
CBAM	Convolutional Block Attention Module
RFA	receptive field attention
PAN-FPN	path aggregation network–feature pyramid network
DFL	Distribution Focal Loss
FPN	feature pyramid network
FPS	frames per second
Grad-CAM	Gradient-weighted Class Activation Mapping
PANet	path aggregation network
ESDNet	Effective Small Object Detection Network
EDF-FAM	Enhanced Dual-Path Feature Fusion Attention Module
FDR	Fine-grained Distribution Refinement
GO-LSD	Global Optimal Localization Self-Distillation
